# Supplementary material for: A magnetically retrievable air and moisture stable gold and palladium nanocatalyst for efficient C−C coupling reactions
Source: R Soc Open Sci. 2020 Sep 30;7(9):200916. doi: 10.1098/rsos.200916 (PMC7540803; doi:10.1098/rsos.200916)
Supplement: Detailed procedures [file rsos200916supp1.docx]

**Supplementary Information**

**A Magnetically Retrievable Air and Moisture Stable Gold and Palladium Nanocatalyst for C-C Coupling Reactions**

Chatura Goonesinghe,^a^ Rivi Ratnaweera ^a^ Mohamed Shaik ^a^ K.M. Nalin de Silva^a^ and Rohini de SIlva*^a^

^a^Centre for Advanced Materials and Devices (CAMD), Department of Chemistry, University of Colombo, Colombo 03, Sri Lanka.
rohini@chem.cmb.ac.lk

**Table of Contents**

[**A.** **Experimental procedures** 2](#_Toc34910587)

[**B.** **Characterization of AuPd@AMNPs** 5](#_Toc34910588)

[**C.** **Mass spectra of products** 9](#_Toc34910589)

[**D.** **References** 13](#_Toc34910590)

## **Experimental procedures**

**General considerations.** The synthesized nanoparticles were analyzed using Fourier Transform Infrared (FTIR) spectroscopy using a Bruker Vertex80 spectrophotometer in the wave number range 400 cm^−1^ and 4000 cm^−1^. Samples were prepared in the form of pellets using KBr, maintaining the KBr : sample mass ratio at 1 : 10. Scanning Electron Microscopy (SEM) images and surface elemental analysis of AMNPs and AuPd@AMNPs was conducted using a Hitachi SU6600 FE-SEM. X-ray diffraction analysis of the synthesized AMNPs and AuPd@AMNPs was performed using a Bruker D8 Focus X-ray powder diffractometer using Cu Kα radiation (= 0.154 nm) over the 2θ range of 5°–80°, with a step size of 0.02° and a step time of 1 s. Elemental analysis of AMNPs and AuPd@AMNPs was done using Inductively Coupled Plasma Mass Spectrometry (ICP MS) using the Agilent 7000 ICP MS System. Samples (25.0 mg were digested in H_2_O_2_ followed by dissolution in a mixture of HCl and HNO_3_, the final volume was made up to 10.0 ml).The products of catalytic reactions were characterized by GC/MS using Agilent 5977A Series GC/MSD System having a 30 m x 250 µm x 0.25 µm HP-5ms column. The temperature program was 40-250 ℃ at 30 ℃ min^-1^ with a final temperature isothermal hold for 12 minutes. The MS mass limit was set between 50 and 450 Da.

**Materials.** All the chemicals were commercially obtained in analytical grade and used without further purification. Gold and palladium decorated amine functionalized magnetite nanoparticles was synthesized by using, iron(III) chloride (FeCl_3_, anhydrous, >99% w/w, Merck Ltd.), 1,6-hexanediamine (The British Drug House Ltd.), sodium acetate anhydrous (Daejung Chemicals & Metals Co.), ethylene glycol (anhydrous 99.8%, Sigma-Aldrich), sodium borohydride (Daejung Chemicals & Metals Co.), palladium(II) chloride (PdCl_2_, 99.9%, metal basis Pd 59.0% min., Alfa Aesar) and gold chloride hydrate (HAuCl_4_, purum, 50% Au, Fluka). Distilled water was used for preparation of all aqueous solutions.The cross coupling reaction were conducted using phenylboronic acid (Sisco Research Laboratories Pvt. Ltd.) and organohalides such as 4-iodotoluene (Alfa Aesar, UK), 4-bromoanisole (>97%, Fluka), 1-bromo-4-fluorobenzene (>97%, Fluka), 4-bromophenol (BDH Chemicals Ltd.), 3-bromobenzotrifluoride (>97%, Fluka). Sodium orthophosphate (≥96%, Sigma-Aldrich) was used as the base in the reaction. Acetonitrile (≥99.8%, Sigma-Aldrich) and diethyl ether (≥99.8%, Sigma-Aldrich) were used as solvents.

**Synthesis of AMNPs.** AMNPs were synthesized using a previously reported solvothermal technique.^1^ A mass of 2.0 g FeCl_3,_ 4.0 g anhydrous sodium acetate and 7.8 mL of 1,6-diaminohexane in 50 ml ethylene glycol was stirred at 45 ℃ for 30 minutes. This homogenized solution was transferred to a steel bomb reactor and maintained at 200 ℃ for 8 hours. The synthesized AMNPs were thoroughly washed with distilled ethanol several times to remove solvent and unreacted 1,6-diaminohexane. The AMNPs were vacuum dried at 45℃ to obtain a black powder.

**Synthesis of Au_50_Pd_50_@AMNPs.** The synthesized AMNPs sample (1.0 g) was dispersed in 50 ml ethanol and ultrasonicated for 30 minutes. To this suspension, HAuCl_4_ (0.33 mmol in solution) and PdCl_2_ (0.33 mmol in solution) was added and the resulting mixture was ultrasonicated again for 60 minutes. Following ultrasonication, the system was flushed using gaseous nitrogen and the solution was stirred under a nitrogen atmosphere for 20 minutes. Next, an excess of cold, 0.01 M sodium borohydride was allowed to drip down slowly on to the mixture with vigorous stirring, under a nitrogen atmosphere. The mixture stirred for two hours to obtain the reduced products, which was separated out using an external magnet. The Pd/Au@AMNPs were thoroughly washed using distilled ethanol.

**Representative Suzuki-Miyaura cross-coupling reaction.** In a round bottomed flask, phenylboronic acid (2.0 mmol) and arylhalide (2.0 mmol) were mixed with 10 ml ACN and stirred for 15 minutes until all solids dissolved. To this mixture, 5.0 ml of Na_3_PO_4_ (10% w/v) was added followed by 100 mg of Au_50_Pd_50_@AMNPs. The nanocatalyst was suspended in solution using ultrasonication for 30 minutes. The solution was heated to reflux and reacted for 4 hours under a N_2_ balloon. The reaction was monitored through thin layer chromatography (TLC) with *n*-pentane was used as the eluting solvent. The mixture was cooled to room temperature and diluted with distilled water (5 mL) and diethyl ether (20 mL). The catalyst was magnetically retained using an external magnet and was washed thrice with ethanol, dried and stored. The organic phase of the reaction mixture was extracted with diethyl ether (3×20 ml) and the combined organics was washed with a % w/v Na_2_CO_3_ solution (20 ml) and brine solution (10 ml) consecutively and dried over anhydrous Na_2_SO_4_. The organic phase was filtered and concentrated under reduced pressure. The concentrated crude product dissolved in diethyl ether and was subjected to GC/MS for characterization.

**Homocoupling of phenylboronic acid.** In a round bottomed flask, phenylboronic acid (2.0 mmol) was mixed with 10 ml ethanol and stirred for 15 minutes until all solids dissolved. To this mixture, 5.0 ml of Na_2_CO_3_ (10% w/v) was added followed by 100 mg of Au_50_Pd_50_@AMNPs. The nanocatalyst was suspended in solution using ultrasonication for 30 minutes. The solution was heated to reflux and reacted for 4 hours under air. The mixture was cooled to room temperature and diluted with distilled water (5 mL) and diethyl ether (20 mL). The catalyst was magnetically retained using an external magnet and was washed thrice with ethanol, dried and stored. The organic phase of the reaction mixture was extracted with diethyl ether (3×20 ml) and the combined organics was washed with a % w/v Na_2_CO_3_ solution (20 ml) and brine solution (10 ml) consecutively and dried over anhydrous Na_2_SO_4_. The organic phase was filtered and concentrated under reduced pressure. The concentrated crude product dissolved in diethyl ether and was subjected to GC/MS for characterization.

**Oxidation of phenylboronic acid to phenol.** In a round bottomed flask, phenylboronic acid (2.0 mmol) was mixed with 10 ml ethanol and stirred for 15 minutes until all solids dissolved. To this mixture, 5.0 ml of Na_2_CO_3_ (10% w/v) was added followed by 100 mg of Au_50_Pd_50_@AMNPs. The nanocatalyst was suspended in solution using ultrasonication for 30 minutes. The solution was heated to reflux and reacted for 4 hours under pure O_2_ gas. The mixture was cooled to room temperature and diluted with distilled water (5 mL) and diethyl ether (20 mL). The catalyst was magnetically retained using an external magnet and was washed thrice with ethanol, dried and stored. The organic phase of the reaction mixture was extracted with diethyl ether (3×20 ml) and the combined organics was washed with a % w/v Na_2_CO_3_ solution (20 ml) and brine solution (10 ml) consecutively and dried over anhydrous Na_2_SO_4_. The organic phase was filtered and concentrated under reduced pressure. The concentrated crude product dissolved in diethyl ether and was subjected to GC/MS for characterization.

**Ullmann homocoupling of 4-iodotoluene.** In a round bottomed flask, 4-iodotoluene (2.0 mmol) was mixed with 10 ml ACN and stirred for 15 minutes until all solids dissolved. To this mixture, 5.0 ml of Na_3_PO_4_ (10% w/v) was added followed by 100 mg of Au_50_Pd_50_@AMNPs. The nanocatalyst was suspended in solution using ultrasonication for 30 minutes. The solution was heated to reflux and reacted for 4 hours under a N_2_ balloon. The mixture was cooled to room temperature and diluted with distilled water (5 mL) and diethyl ether (20 mL). The catalyst was magnetically retained using an external magnet and was washed thrice with ethanol, dried and stored. The organic phase of the reaction mixture was extracted with diethyl ether (3×20 ml) and the combined organics was washed with a % w/v Na_2_CO_3_ solution (20 ml) and brine solution (10 ml) consecutively and dried over anhydrous Na_2_SO_4_. The organic phase was filtered and concentrated under reduced pressure. The concentrated crude product dissolved in diethyl ether and was subjected to GC/MS for characterization.

**Hydrogenation of 1-Chloro-3-nitrobenzene.** In a round bottomed flask, 1-Chloro-3-nitrobenzene (2.0 mmol) was mixed with 10 ml ethanol and stirred for 15 minutes until all solids dissolved. To this mixture, 100 mg of Au_50_Pd_50_@AMNPs was added and suspended in solution using ultrasonication for 30 minutes. The solution was heated to reflux and reacted for 4 hours under H_2_ gas. The mixture was cooled to room temperature and diluted with distilled water (5 mL) and diethyl ether (20 mL). The catalyst was magnetically retained using an external magnet and was washed thrice with ethanol, dried and stored. The organic phase of the reaction mixture was extracted with diethyl ether (3×20 ml) and the combined organics was washed with a % w/v Na_2_CO_3_ solution (20 ml) and brine solution (10 ml) consecutively and dried over anhydrous Na_2_SO_4_. The organic phase was filtered and concentrated under reduced pressure. The concentrated crude product dissolved in diethyl ether and was subjected to GC/MS for characterization.

## **Characterization of AuPd@AMNPs**


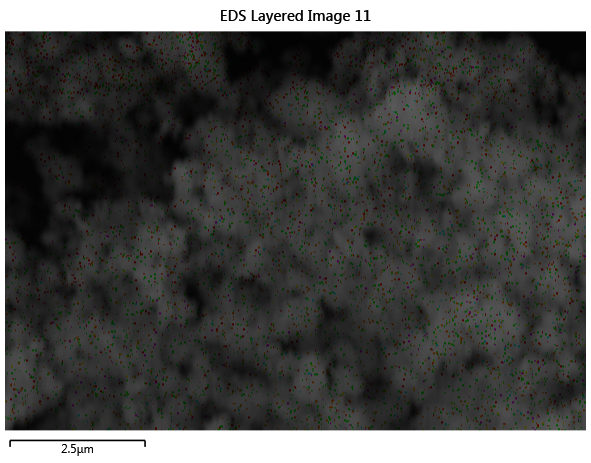


**Figure S1** EDS layered image of AMNPs


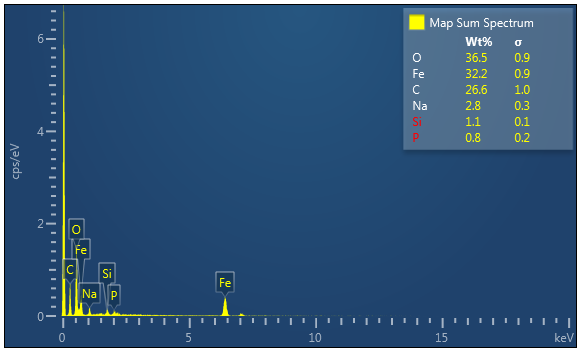


**Figure S2** EDS spectrum of AMNPs


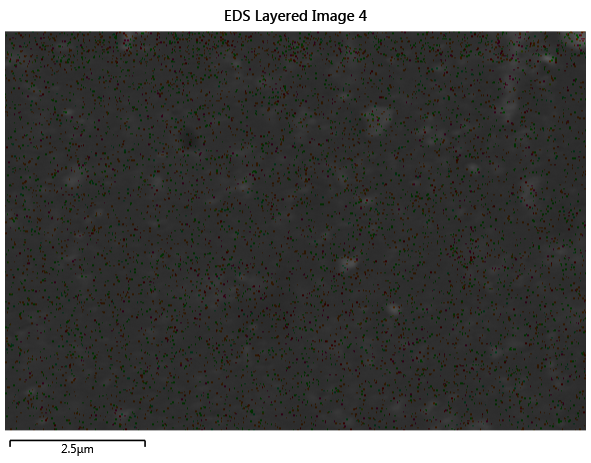


**Figure S3** EDS layered image of Au_50_Pd_50_@AMNPs


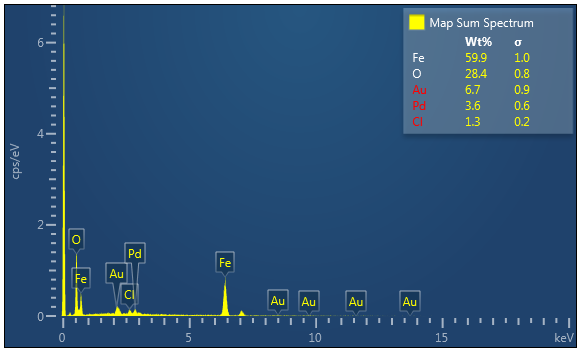


**Figure S4** EDS spectrum of Au_50_Pd_50_@AMNPs


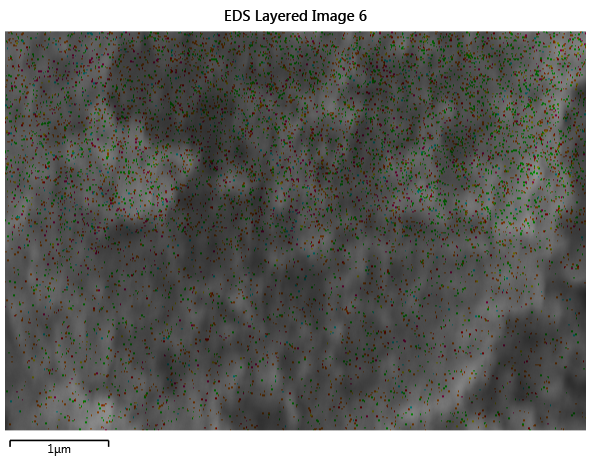


**Figure S5** EDS layered image of post-reaction Au_50_Pd_50_@AMNPs


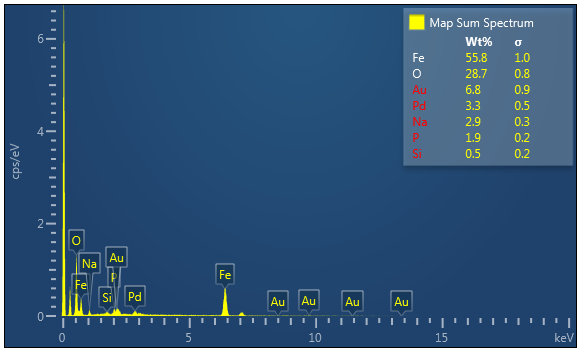


**Figure S6** EDS spectrum of post-reaction Au_50_Pd_50_@AMNPs

**Table S1** Gold and palladium composition analysis of AuPd@AMNPs

| **Entry** | **Catalyst** | **Initial mmol/g** | | **mg/g** | | **mmol/g** | | **w/w%** | |
| --- | --- | --- | --- | --- | --- | --- | --- | --- | --- |
|  |  | **Pd** | **Au** | **Pd** | **Au** | **Pd** | **Au** | **Pd** | **Au** |
| 1 | AMNP | - | - | 0.00 | 0.00 | 0.00 | 0.00 | 0.00 | 0.00 |
| 2 | Au_0_Pd_100_@AMNP | 0.667 | 0.00 | 7.16 | 0.00 | 0.07 | 0.00 | 0.71 | 0.00 |
| 3 | Au_25_Pd_75_@AMNP | 0.500 | 0.167 | 12.53 | 12.90 | 0.12 | 0.07 | 1.25 | 1.29 |
| 4 | Au_50_Pd_50_@AMNP | 0.333 | 0.333 | 11.92 | 37.37 | 0.11 | 0.19 | 1.19 | 3.73 |
| 5 | Au_75_Pd_25_@AMNP | 0.167 | 0.500 | 3.73 | 55.27 | 0.04 | 0.28 | 0.37 | 5.53 |
| 6 | Au_90_Pd_10_@AMNP | 0.067 | 0.600 | 1.35 | 51.85 | 0.01 | 0.26 | 0.14 | 5.18 |
| 7 | Au_100_Pd_0_@AMNP | 0.000 | 0.667 | 0.00 | 61.90 | 0.00 | 0.31 | 0.00 | 6.19 |
| 8 | Post reaction | - | - | 11.50 | 37.24 | 0.11 | 0.19 | 1.15 | 3.72 |
| 9 | 6 cycles | - | - | 9.88 | 28.83 | 0.09 | 0.15 | 0.99 | 2.88 |

## **Mass spectra of products**


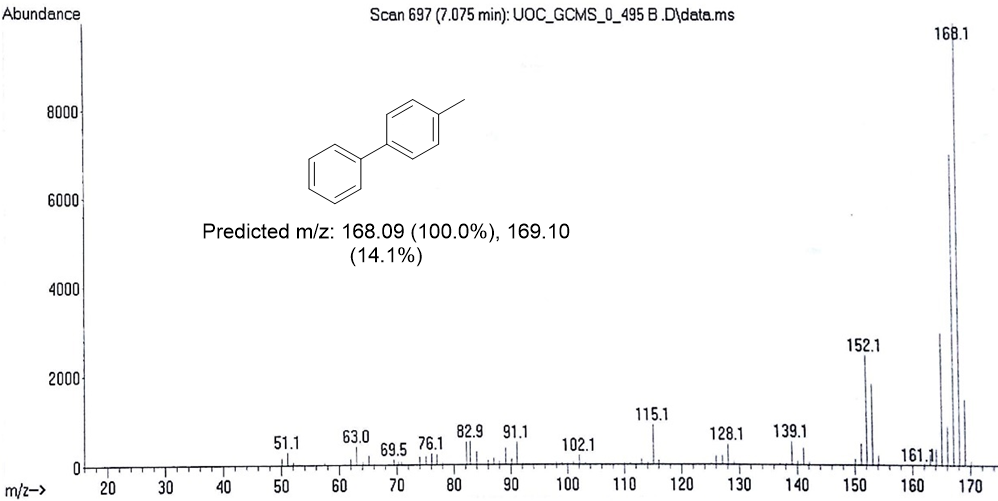


**Figure S7** Mass spectrum obtained for 4-phenyltoluene


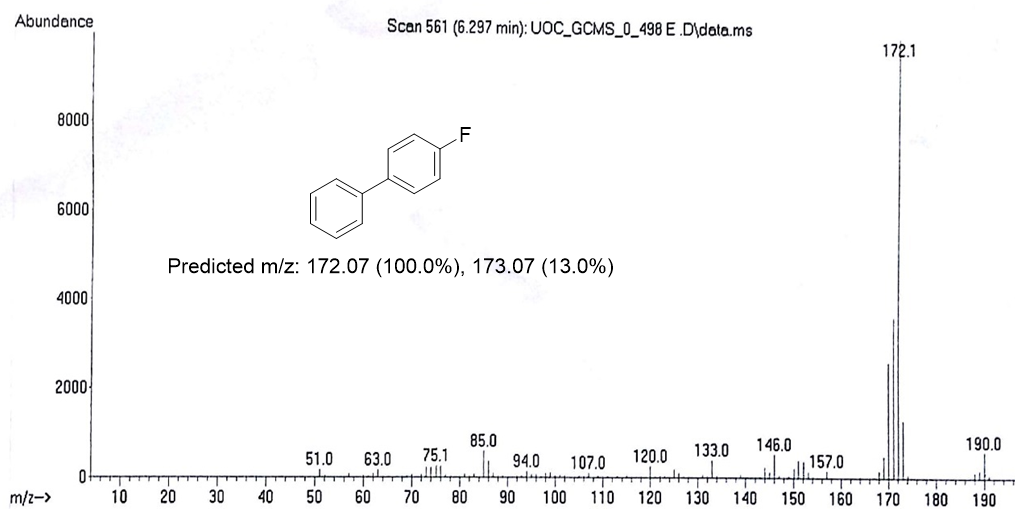


**Figure S8** Mass spectrum obtained for 4-fluorobiphenyl


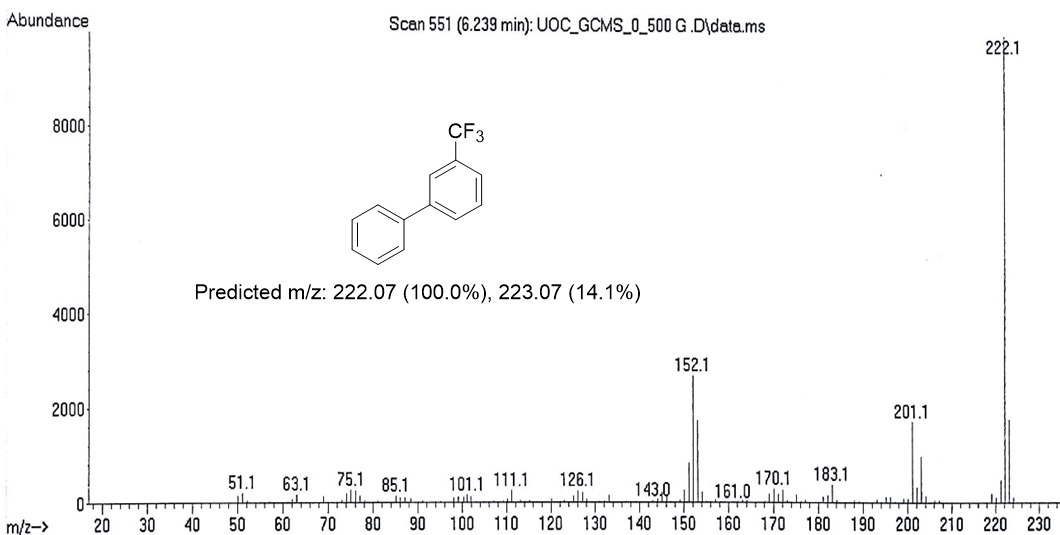


**Figure S9** Mass spectrum obtained for 3-(trifluoromethyl)-1,1'-biphenyl


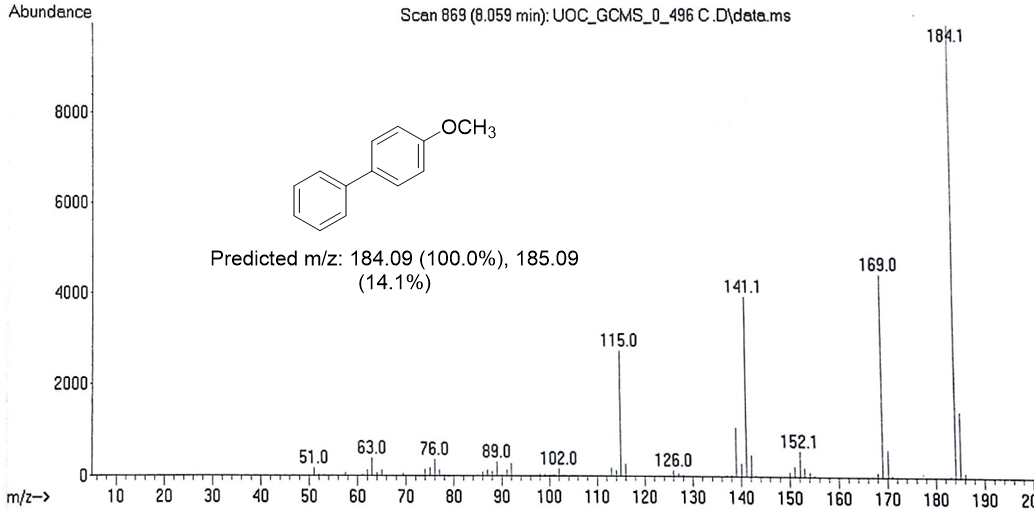


**Figure S10** Mass spectrum obtained for 4-phenylanisole


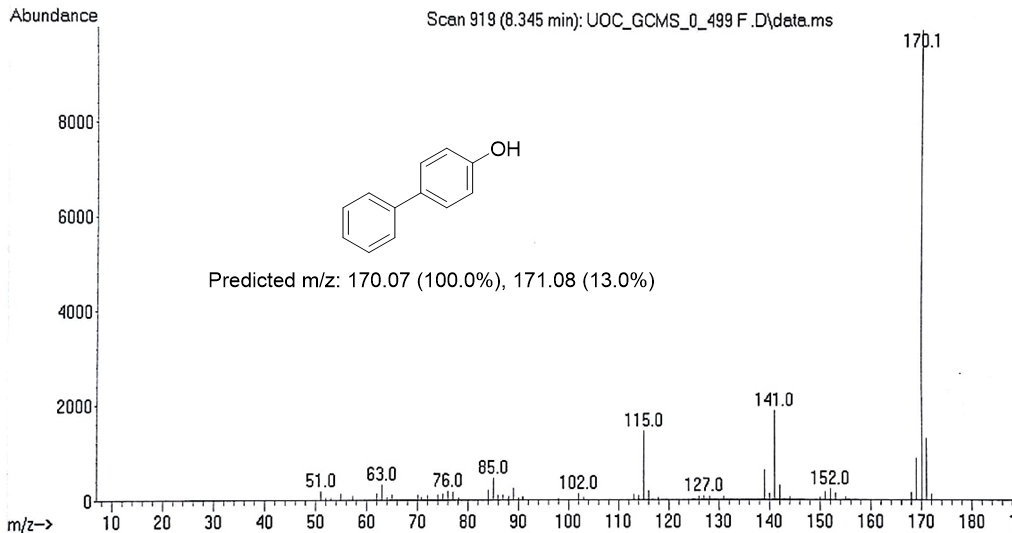


**Figure S11** Mass spectrum obtained for 4-phenylphenol


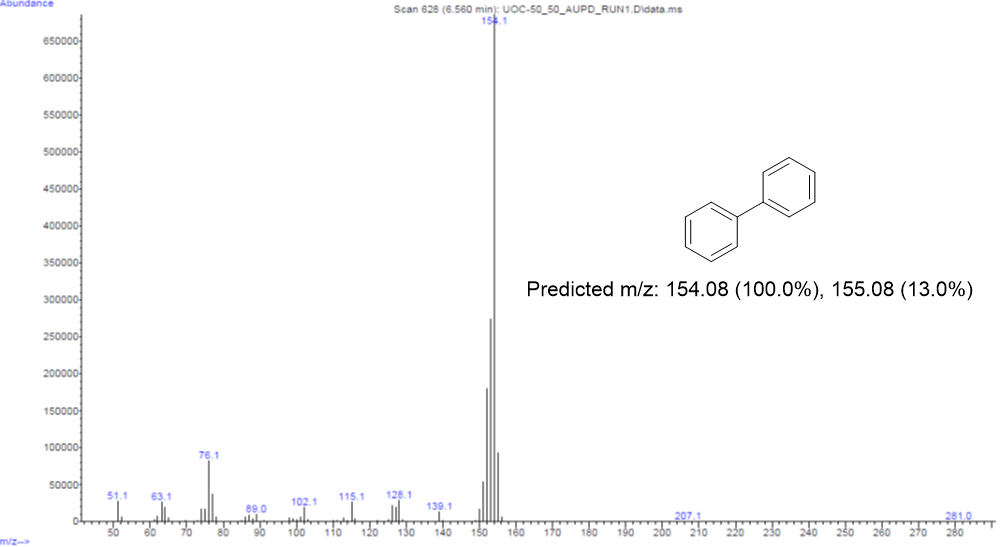


**Figure S12** Mass spectrum obtained for biphenyl


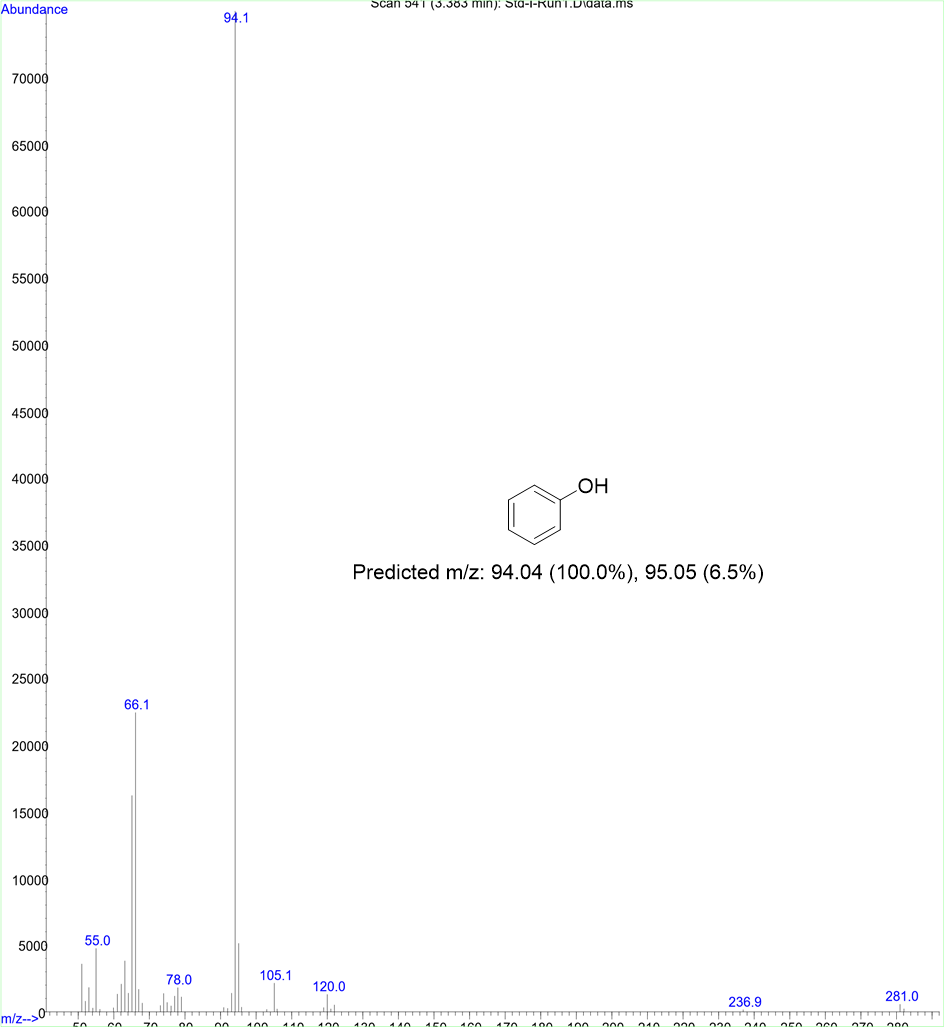


**Figure S13** Mass spectrum obtained for phenol


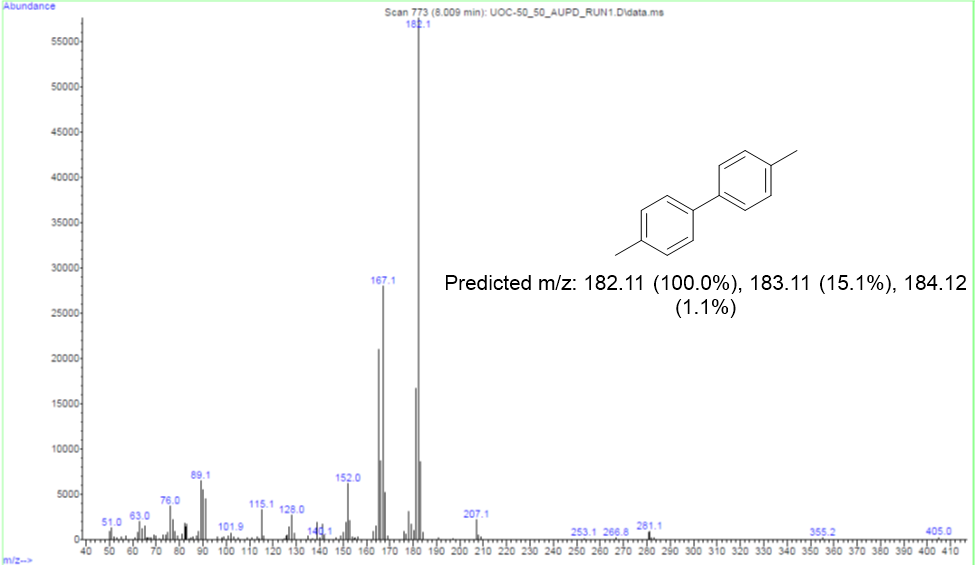


**Figure S14** Mass spectrum obtained for 4,4'-dimethyl-1,1'-biphenyl

## **References**

(1) Wang, L.; Bao, J.; Wang, L.; Zhang, F.; Li, Y. One-Pot Synthesis and Bioapplication of Amine-Functionalized Magnetite Nanoparticles and Hollow Nanospheres. *Chem. Eur. J.* **2006**, *12* (24), 6341–6347.
